# Platinum-based chemotherapy promotes antigen presenting potential in monocytes of patients with high-grade serous ovarian carcinoma

**DOI:** 10.3389/fimmu.2024.1414716

**Published:** 2024-09-09

**Authors:** Irina Larionova, Pavel Iamshchikov, Anna Kazakova, Militsa Rakina, Maxim Menyalo, Kadriia Enikeeva, Guzel Rafikova, Yuliya Sharifyanova, Valentin Pavlov, Alisa Villert, Larisa Kolomiets, Julia Kzhyshkowska

**Affiliations:** ^1^ Laboratory of Translational Cellular and Molecular Biomedicine, National Research Tomsk State University, Tomsk, Russia; ^2^ Cancer Research Institute, Tomsk National Research Medical Center, Russian Academy of Sciences, Tomsk, Russia; ^3^ Institute of Urology and Clinical Oncology, Bashkir State Medical University of the Ministry of Health of Russia, Ufa, Russia; ^4^ Institute of Transfusion Medicine and Immunology, Institute for Innate Immunoscience (MI3), Medical Faculty Mannheim, University of Heidelberg, Mannheim, Germany; ^5^ German Red Cross Blood Service Baden-Württemberg – Hessen, Mannheim, Germany

**Keywords:** monocyte, ovarian cancer, chemotherapy, single cell sequencing, transcriptome, methylation, antigen presentation

## Abstract

Ovarian cancer (OC) is the most lethal gynecologic malignancy worldwide. The major clinical challenge includes the asymptomatic state of the disease, making diagnosis possible only at advanced stages. Another OC complication is the high relapse rate and poor prognosis following the standard first-line treatment with platinum-based chemotherapy. At present, numerous clinical trials are being conducted focusing on immunotherapy in OC; nevertheless, there are still no FDA-approved indications. Personalized decision regarding the immunotherapy, including immune checkpoint blockade and immune cell–based immunotherapies, can depend on the effective antigen presentation required for the cytotoxic immune response. The major aim of our study was to uncover tumor-specific transcriptional and epigenetic changes in peripheral blood monocytes in patients with high-grade serous ovarian cancer (HGSOC). Another key point was to elucidate how chemotherapy can reprogram monocytes and how that relates to changes in other immune subpopulations in the blood. To this end, we performed single-cell RNA sequencing of peripheral blood mononuclear cells (PBMCs) from patients with HGSOC who underwent neoadjuvant chemotherapeutic treatment (NACT) and in treatment-naïve patients. Monocyte cluster was significantly affected by tumor-derived factors as well as by chemotherapeutic treatment. Bioinformatical analysis revealed three distinct monocyte subpopulations within PBMCs based on feature gene expression – CD14.Mn.S100A8.9hi, CD14.Mn.MHC2hi and CD16.Mn subsets. The intriguing result was that NACT induced antigen presentation in monocytes by the transcriptional upregulation of MHC class II molecules, but not by epigenetic changes. Increased MHC class II gene expression was a feature observed across all three monocyte subpopulations after chemotherapy. Our data also demonstrated that chemotherapy inhibited interferon-dependent signaling pathways, but activated some TGFb-related genes. Our results can enable personalized decision regarding the necessity to systemically re-educate immune cells to prime ovarian cancer to respond to anti-cancer therapy or to improve personalized prescription of existing immunotherapy in either combination with chemotherapy or a monotherapy regimen.

## 1 Introduction

Ovarian cancer (OC) is the most lethal gynecologic malignancy in the world (1). In 2022 there were 324,603 incidences of OC and 206,956 deaths worldwide (2). According to the Globocan’s 2022 projections, by the year 2040 incidence will have risen by 42% to a total of just over 446,000, with an even larger increase in the number of deaths each year (up nearly 52% to over 314,000) (3). Most patients with ovarian cancer are asymptomatic and are often diagnosed at advanced stages. This has led to OC being labeled as the “silent killer” (1). The principal hallmark of advanced-stage OC is ascites — the accumulation of excessive fluid containing cellular and acellular components in the abdomen (4). Advanced stages of OC are associated with poor prognosis and a significant decrease in survival rate compared to those diagnosed at stage I, although survival rate may vary according to the different disease histotypes (5). High-grade serous ovarian cancer (HGSOC) accounts for approximately 70% of all cases, making it the most common and deadliest histotype (1).

Most OC patients undergo primary debulking surgery combined with platinum/taxan-based chemotherapy, with or without the angiogenesis inhibitor bevacizumab, and, in certain cases, followed by maintenance treatment with poly-ADP-ribose polymerase (PARP) inhibitors (6, 7). However, if complete cytoreduction cannot be achieved by debulking surgery, an alternative therapeutic option for these patient groups is neoadjuvant chemotherapy (NACT) (8). Despite a good response to standard first-line chemotherapy, relapse occurs in 70% of patients within a short period of time (4, 6).

The tumor microenvironment (TME) in ovarian cancer is complex and unique, containing multiple cell types populating both fluid (ascites) and solid (omentum) niches (4, 7). TME targeting in ovarian cancer is intensely developing and the main focus is on immune cells, cancer-associated fibroblasts, endothelial cells and ECM-tumor cell interactions (7, 9–11). Among cells of innate immune system, tumor-associated macrophages (TAMs) are the most abundant cell population in the TME. TAMs may facilitate tumor growth, activate angiogenesis, induce immunosuppression and mediate chemoresistance (12, 13). The major plastic source for TAMs are peripheral blood monocytes (14, 15). Accumulating evidence suggests that the tumor-induced systemic environment can re-program monocytes before their arrival to the tumor site (14). Several studies reported transcriptional alterations in circulating monocytes in several cancers, including human breast cancer (16, 17), colorectal cancer (18, 19), renal cancer (20), and hepatic cancer (21). Role of circulating monocytes in ovarian cancer progression and response to chemotherapeutic intervention remains undefined which indicates high relevance of all attempts to uncover pro- and anti-tumor phenotypes of monocytes.

In our study we present the results of single-cell RNAseq analysis of peripheral blood mononuclear cells (PBMCs) from patients with HGSOC. One of the major aims of our study was to evaluate the effects of chemotherapy on the peripheral immune system, and to this end we compared samples of patients who were treated with neoadjuvant chemotherapy (NACT) before surgery and treated only by standard care therapy (debulking surgery and adjuvant chemo(targeted) therapy). We focused on monocyte population as it was one of the most altered by both cancer-derived systemic factors and NACT subset.

## 2 Materials and methods

### 2.1 Clinical samples

The study group consisted of 10 cases of advanced stage HGSOC (IIIС stage) diagnosed and treated in the Cancer Research Institute, Tomsk National Research Medical Centre (Tomsk, Russia). Patients had no acute pathologies, no infectious disorders, and did not have a history of any other types of cancer in addition to HGSOC. All 10 samples were used to conduct reduced representation bisulfite sequencing (RRBS) and 6 samples were used for single-cell RNA sequencing. Study group was divided according to treatment plan: 3 patients received platinum-based neoadjuvant chemotherapy (NACT) [carboplatin plus paclitaxel] prior to surgery and 3 patients had debulking surgery without upfront NACT. After surgery all patients received platinum/taxane-based adjuvant chemotherapy.

Healthy volunteers (N=10) were enrolled in this study as a control group. The inclusion criteria for the healthy cohort were: no active medical conditions, and no current or past history of an oncology disease.

The study received approval from the Local Committee for Medical Ethics and was conducted in accordance with the guidelines of the Declaration of Helsinki and the International Conference on Harmonization Good Clinical Practice Guidelines (ICH GCP). Written informed consent was obtained from all subjects, including the patients/participants, who willingly provided their consent to participate in the study.

### 2.2 Monocyte isolation

Peripheral whole-blood samples were collected from patients with ovarian cancer (n=10) and healthy donors (n=10). The peripheral blood mononuclear cells (PBMCs) were separated from whole blood by density gradient centrifugation using Lymphosep, Lymphocyte Separation Media (#L0560-500, Biowest, France), 1.077 g/ml density, at 600g for 30 minutes. The isolation of monocytes from PBMCs was performed using positive magnetic selection with CD14+ MACS beads (#130-050-201, Miltenyi Biotech, Germany), resulting in 90–98% monocyte purity as confirmed by flow cytometry. After monocyte isolation, the samples were washed twice with DPBS without calcium and magnesium at 300 g for 10 minutes. Subsequently, the cell precipitate was lysed using lysis buffer RLT (#79216, Qiagen, USA) and stored at -80°C until further experiments. Lysed monocytes were used for DNA isolation and reduced representation bisulfite sequencing (RRBS-Seq).

### 2.3 PBMC preparation for single-cell sequencing

PBMCs were washed twice with DPBS without calcium and magnesium at 300 g for 10 minutes and counted using CytoFLEX flow cytometer (Beckman Coulter, USA). 1×10^6^ PBMCs were used to prepare cell suspension for single-cell sequencing. Cells were put into 1.5 ml cryo tubes and mixed with 500 μl of X-VIVO™ 10 Medium (#180989, Lonza, Switzerland), 400 μl of fetal bovine serum (#10500064, ThermoFisher Scientific, USA), and 100 μl of dimethyl sulfoxide (#F135, Paneco, Russia). Subsequently, the cell suspension was stored in cryo container at -80°C for 48 hours, and then stored in liquid nitrogen until further experiments (but not more than 6 months).

### 2.4 Single-cell sequencing

Single-cell RNA sequencing was performed on the Chromium X platform, using the 10х Genomics Chromium Next GEM Single Cell 3′ Reagent Kit v3.1. (10х Genomics, USA). Prior to library preparation, cells were counted and quality of samples was assessed. Up to 8000 cells were used for further manipulations. cDNA amplification and library construction were conducted following the manufacturer’s protocol. Sequencing was performed with the Illumina Nextseq 2000 platform (Illumina, USA).

### 2.5 Single-cell RNAseq data analysis

Basic processing of raw sequencing data was performed in Cell Ranger 7.1.0 (22) using human genome reference GRCh38-2020-A with default parameters. Resulting gene-barcode matrices were analyzed in Seurat (23) in R environment. Doublet cells effect was addressed with scDblFinder (24). SCTransform normalization was used to normalize raw counts. Linear dimension reduction was performed with PCA. Batch correction was performed using Harmony. First 40 Harmony corrected principal components were used in clustering and non-linear dimension reduction via UMAP in Seurat. Resulting cell clusters were annotated using SingleR (25) with Monaco et al. reference (26) and manual annotation with Human Protein Atlas scRNAseq human blood atlas (27). Differential gene expression analysis between sample groups was conducted with pseudo-bulk approach (28). Thus, individual cell clusters having not less than 45 cells and 1000 total raw counts were sample-wise aggregated using sum of raw counts. Batch-effect was corrected using ComBat-seq (29), and differential expression analysis was conducted in DESeq2 package (30). Gene set enrichment analysis (GSEA) was carried out with fgsea package (31). Individual cell clusters having not less than 45 cells and 1000 total raw counts were sample-wise aggregated using sum of raw counts. AUGUR tool (32) was used to indicate most responsive cells to biological perturbations in single-cell data. Compositional data analysis was performed with scCODA tool (33) to analyze changes in cell abundancies. UCell packages (34) was used to evaluate gene signatures distribution among cell clusters and sample groups. Cell-cell communication was profiled with ligand-receptor interaction analysis in Liana tool (35). Gene coexpression networks in monocytes were analyzed with hdWGCNA package (36). Co-expression modules were analyzed with fgsea package to evaluate enrichment of modules in different samples groups. Visualization was performed using Seurat, scCustomize (37), Cpubr (38), EnhancedVolcano (39) and ggplot2 (40).

### 2.6 DNA extraction

DNA were extracted from lysed monocyte samples using AllPrep DNA/RNA/miRNA Universal Kit (#80224, Qiagen, USA). The quality of DNA was assessed by TapeStation 4150 automated electrophoresis system (#RRID: SCR_019393, Agilent Technology, USA). The quantity of DNA was assessed by Qubit 4 fluorometer (#RRID: SCR_018095, ThermoFisher Scientific, USA).

### 2.7 Reduced representation bisulfite sequencing

Samples were purified using magnetic beads AMPure XP (Beckman Coulter) and concentration was controlled using Fluoroskan. Libraries were prepared using Zymo-Seq RRBS Library Kit (#D5461) and Zymo-Seq UDI Primer Plate (#D3096) (Zymo Research, USA), according to the manufacturer’s instructions. Sequencing was performed by the Illumina NovaSeq 6000 platform (Illumina, USA).

### 2.8 Reduced representation bisulfite sequencing data analysis

RRBS sequencing data processing included quality control and trimming of technical sequences, mapping of reads to the reference genome, counting the level of cytosine methylation in the CpG context, and differential methylation analysis. Quality control and trimming of adapter sequences were performed in FastQC programs (41) and Trim_Galore (42). Mapping of RRBS reads to the human reference genome GRCh38 was performed using the Bismark program (43). Cytosine methylation levels in CpG context were also counted in Bismark. Differential methylation analysis was performed in the R environment, using the edgeR package (44), negative binomial modeling of raw methylation counts to search for differentially methylated CpG sites was performed following this manual (45). We obtained a list of genes with hyper- and hypomethylated CpG sites in proximity of promoter regions (-10000 and +10000 bp from transcription start site), and enriched the obtained gene list by biological pathways in the online tool Enrichr (46).

## 3 Results

### 3.1 Single cell RNAseq analysis revealed tumor-specific re-programming of peripheral blood monocytes

**Figure 1:**
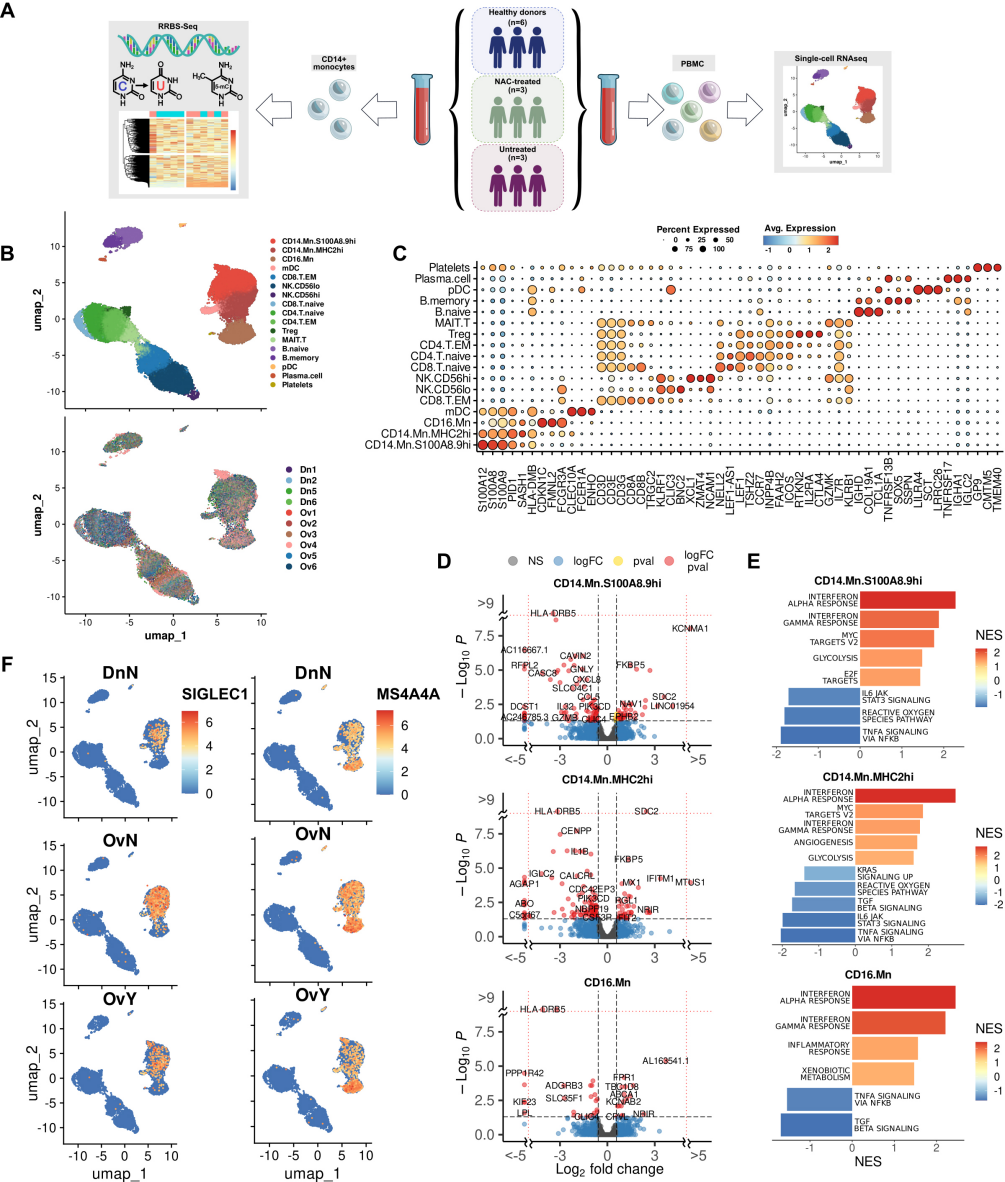
Single cell RNAseq analysis of PBMCs in high-grade serous ovarian carcinoma revealed tumor-specific programming of monocytes **(A)** Overall study design. **(B)** Distribution of PBMC cell clusters and individual samples in UMAP 2D dimensions. **(C)** Dot plot discriminates marker genes for each cluster. **(D)** Volcano plots demonstrate p-value and log2fold-change value for DEGs in each monocyte subpopulation (CD14.Mn.S100A8.9hi; CD14.Mn.MHC2hi; CD16.Mn) versus healthy control (|L2FC|>0.58, FDR<0.05). **(E)** Bar plots with GSEA results indicate top deregulated pathways in each monocyte subpopulation (CD14.Mn.S100A8.9hi; CD14.Mn.MHC2hi; CD16.Mn) versus healthy control (FDR<0.1). **(F)** Localization of *SIGLEC1* and *MS4A4A* gene expression among individual cells on UMAP. DnN – healthy control (donor group), OvN – treatment-naive group, OvY – NAC-treated group.

PBMCs were obtained from six patients with high-grade serous ovarian carcinoma (HGSOC) and six healthy donors (**Figure 1A**). Blood samples were collected after 2-3 courses of platinum-based neoadjuvant chemotherapy (NACT) for three patients and after surgery without NACT courses for other three cases. All patients underwent adjuvant chemotherapy (paclitaxel+carboplatin) after surgery. Isolated PBMCs were analyzed on a 10x Chromium platform, and the transcriptome of each sample was obtained using Illumina NextSeq 2000 platform. Raw sequencing reads were processed using Cell Ranger 7.1.0 (look material and methods section) to perform quality control, read alignment in individual cells, and count gene-barcode matrix. Resulting gene-barcode matrices were processed via Seurat package in R environment. Cells with less than 300 detected genes, 1000 UMIs, 5% of ribosomal transcripts, and more than 10% of mitochondrial transcripts were filtered out as low-quality cells. Removal of cell doublets was addressed with scDblFinder tool. Raw counts normalization was performed via SCTransform for further PCA and UMAP dimension reduction and Louvain clustering in Seurat. A total of 28,779 high-quality cells and 17 cell clusters were obtained for analysis. Two samples obtained from healthy donors failed quality control (QC) and were excluded in the further analysis. UMAP plots of PBMCs derived from treated and untreated patients with HGSOC (n = 6) and healthy donors (n = 4) are shown in **Figure 1B**. The PBMC clusters were annotated automatically using SingleR tool with Monaco et al. reference and manually using Human Protein Atlas human blood single-cell atlas (27). Concordant result was used as final annotation comprising following known cell lineages: T/NK cells (Treg; CD4.T.naive; CD8.T.naive; CD4.T.EM; MAIT.T.; CD8.T.EM; NK.CD56lo; NK.CD56hi), myeloid cells (CD14.Mn.S100A8.9hi; CD14.Mn.MHC2hi; CD16.Mn; mDC), B-cells (B.naïve; B.memory; Plasma.cell), pDC and Platelets (**Figure 1B**). Top marker genes for each cell type cluster are indicated in Dot plot (**Figure 1C**).

We next proceeded with detailed analysis of monocyte cell cluster to elucidate the differences in monocyte profile between the tumor and healthy states. In order to detect differentially expressed genes between three groups, we used pseudo-bulk approach with DESeq2. The analysis revealed the tumor-specific functional activation of the following processes: cell surface interactions at vascular wall, glycolysis, MTORC1 signaling, serine/threonine kinase activity, angiogenesis, interferon alpha/beta signaling, and interferon gamma signaling. Among up-regulated genes there were *SDC2, FKBP5, MX1, IFITM1, DAPK1, FABP5, SIGLEC1, CEBPD, ABCA1, AREG, CD63*, and others (**Supplementary Figure S1A**). The most significant down-regulated genes included *HLA-DRB5, HLA-DQA2, LRAR1, CXCL8, IL1B, JUNB, CCL5, GZMB, TNF*, and *IL32* related to MHC II protein complex, chemokine and cytokine activity, LPS-mediated signaling, TLR signaling, neutrophil migration and chemotaxis, and antigen binding (**Supplementary Figure S1B**). We concluded that tumor induces monocyte reprogramming towards activating monocyte interaction with vascular wall and suppressing antigen presentation and immune-inflammatory state.

We then questioned whether it can be related to variability in gene patterns for distinct monocyte subpopulations. Three main subpopulations of monocytes were assigned based on the distinct marker profile: CD14.Mn.S100A8.9hi, CD14.Mn.MHC2hi and CD16.Mn (**Figures 1B, C**). We investigated tumor-related changes in each monocyte subset separately. In CD14.Mn.S100A8.9hi cluster, cancer-specific genes of interest included *SIGLEC1, MX1, FKBP5, AREG, HBEGF, LYZ, OAS1, CEBPD, FABP5, MARCO, CD163, DAPK1*, and *PECAM1* among others, related to interferon alpha/gamma pathway, glycolysis, MTORC1 signaling, hypoxia, endocytosis receptor activity, and serine/threonine kinase activity (**Figures 1D, E**). In CD14.Mn.MHC2hi cluster the up-regulation of the following genes of interest was detected in cancer: *SDC2, MS4A4A, MX1, FKBP5, OAS1, CEBPD, FABP5, DAPK1, CD63, IFITM1, VSIG4*, and *CXCR4* annotated with interferon alpha/gamma, MYC targets, angiogenesis, glycolysis, oxidative phosphorylation, MTORC1 signaling, and HSF1 activation. Among suppressed pathways there are inflammation, neutrophil chemotaxis, antimicrobial activity, and TGFb-signaling (**Figures 1D, E**). CD16.Mn cluster was less represented in amount with activated genes in cancer samples. Higher expression of *HIF1A, ABCA1, FPR1, CD63*, and *FCN1* was found in cancer compared to healthy state (**Figures 1D, E**).

Several up-regulated genes, i.e. *SIGLEC1* and *MS4A4A* were indicative only for monocyte cluster within whole PBMC population (**Figure 1F**). We focused on two functionally attractive for monocyte/macrophage lineage cell genes – *SIGLEC1* and *MS4A4A*. Herewith *SIGLEC1* is specifically expressed in CD14.Mn.S100A8.9hi cluster, and *MS4A4A* was significantly indicative for CD14.Mn.MHC2hi cluster (**Figure 1F**).

In total, the presence of cancer induced general pro-tumor reprogramming of monocytes by activating interferon pathways, angiogenesis, glycolysis, oxidative phosphorylation, and lipid metabolism. These changes were strongly indicative for S100A8.9hi and MHC2hi subsets, but in less extend for CD16 subset.

### 3.2 Platinum-based chemotherapy induces monocyte transcriptional programming that differs from overall tumor-specific changes in monocyte profile

In advanced ovarian cancer NACT is a treatment strategy that favors optimal cytoreduction and decreases rates of perioperative morbidity. Despite the high response rate to NACT, tumor progression after or during NACT courses remains an unresolved challenge (8, 47). There is still no indication for immunotherapy for HGSOC patients treated or untreated with NACT, and the lack of effective molecular and cellular targets justifies the intensive investigations of immune-related mechanisms (48). The data on monocyte reprogramming under the chemotherapy in cancer is limited (15). To understand whether chemotherapy can affect monocyte profile in ovarian cancer, we performed comparative analysis of a) NACT-treated PBMC samples with untreated PBMCs and b) NACT-treated PBMC samples with healthy donors` samples.

**Figure 2:**
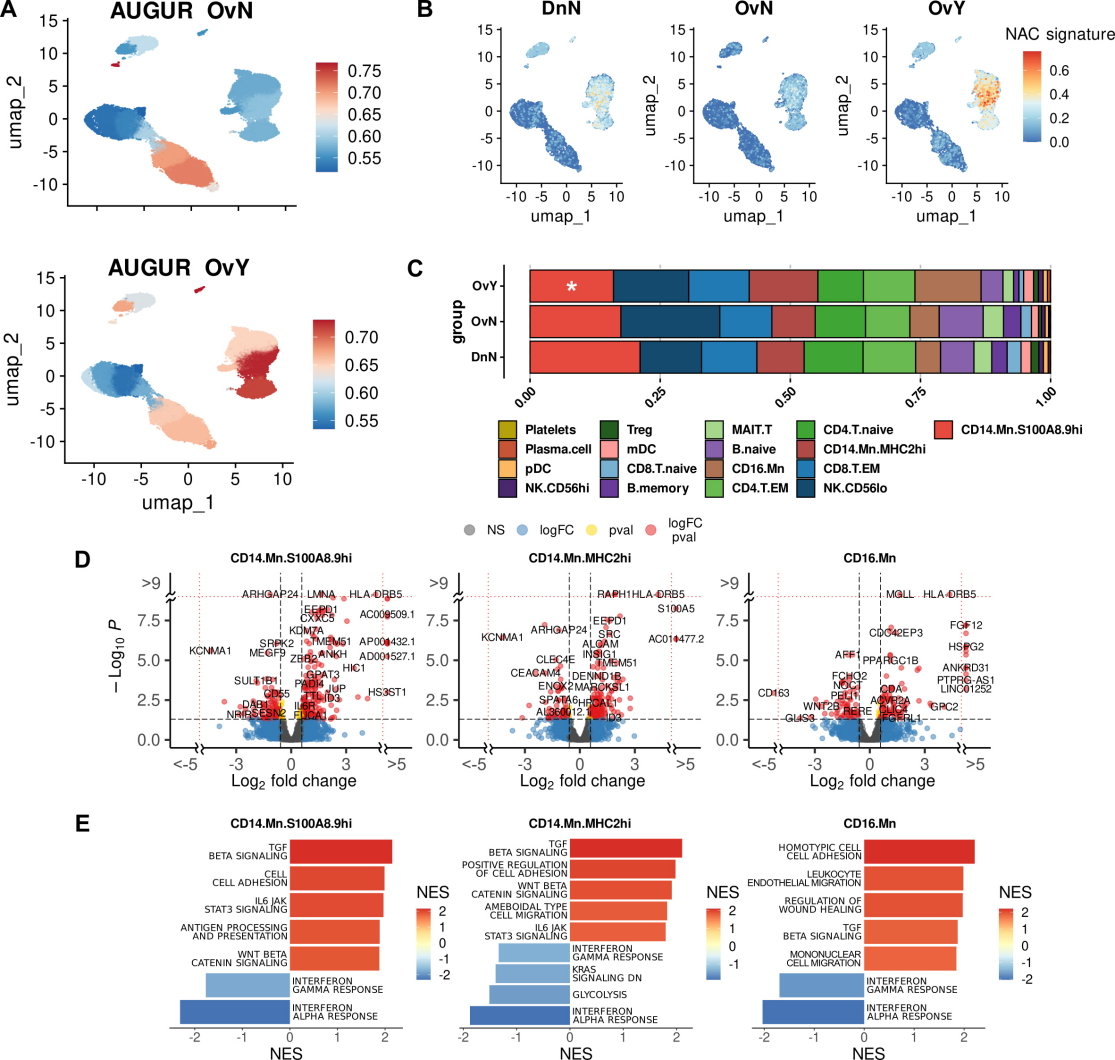
Chemotherapy-induced transcriptional programming in cancer monocytes **(A)** Augur demonstrates top-affected cell types in naive tumor PBMCs (upside) and in NAC-treated PBMCs (underside) (indicated by Augur score). **(B)** NAC-related gene signature obtained using UCell tool. **(C)** Abundance plot demonstrated cell content in studied cohorts. *Significant difference in cell abundance in NAC vs. Dn (FDR<0.1). **(D)** Volcano plots demonstrate p-value and log2fold-change value for DEGs in each monocyte subpopulation (CD14.Mn.S100A8.9hi; CD14.Mn.MHC2hi; CD16.Mn) of NAC-treated monocytes versus healthy control (|L2FC|>0.58, FDR<0.05). **(E)** Bar plots with GSEA results indicate top deregulated pathways in each monocyte subpopulation (CD14.Mn.S100A8.9hi; CD14.Mn.MHC2hi; CD16.Mn) of NAC-treated monocytes versus healthy control (FDR<0.1). DnN – healthy control (donor group), OvN – treatment-naive group, OvY – NAC-treated group.

First, we questioned which cell types are the most responsive to NACT-dependent effects in our single-cell data. We applied Augur, a method to prioritize the cell types most responsive to biological perturbations in a multidimensional space of single-cell data (32). Augur employs a machine-learning framework abolished the dependence on the total number of cells. Mathematically, this method uses area under the receiver operating characteristic curve (AUC) accounting both the amount and magnitude of simulated differential expression. According to this method, cell subsets in NACT-treated samples where the Augur score increased significantly (Augur score more than 0,7) compared to healthy control, included pDC [0,731], CD14.Mn.MHC2hi [0,726], and CD16.Mn [0,715] (**Figure 2A**). When comparing untreated samples with healthy controls prioritized cell populations included Plasma.cell [0,768] and NK.CD56lo [0.715] (**Figure 2A**). In compliance with this algorithm, monocyte cluster undergoes a more extensive transcriptional reprogramming particularly under NACT rather than just in the presence of the tumor overall.

Further in-depth bioinformatic analysis confirmed that chemotherapy affects the monocyte transcriptome more intensely than the tumor-related systemic influence from untreated samples. In NACT-treated patients, there were 241 (FDR < 0.05) activated genes in total in the monocyte cluster compared to healthy donors. This is more than six times the number of activated genes in untreated cancer samples versus healthy controls (39 genes, FDR < 0.05). The most interesting genes included *TMEM51, NOTCH1, EEPD1, MAFB, HLA-DRB5, LRPAP1, TSNARE1, HDAC9, SNAI3, SEMA4A, MARCO, CD63, LRPAP1, ABCA1*, and *HLA-DQB2* among others, annotated with lipid metabolism, NOTCH signaling, cellular response to low-density lipoproteins, mononuclear cell differentiation, lymphocyte/leukocyte differentiation, regulation of Ras protein signal transduction, regulation of small GTPase mediated signal transduction, leukocyte transendothelial migration, oxidative phosphorylation, VEGF signaling, adhesion molecule binding, MTORC1 signaling, and WNT beta catenin signaling (**Supplementary Figures S2A, B**). Such functional pathways like TLR receptor signaling, TNF signaling pathway, complement activation, humoral immune response, and cellular amino acid catabolic processes were suppressed in monocytes under NACT. In monocyte subpopulations (CD14.Mn.S100A8.9hi, CD14.Mn.MHC2hi and CD16.Mn) there were some specific changes under NAC compared to healthy control monocytes (**Supplementary Figures S2C, D**).

When comparing NACT-treated samples with untreated ones in cancer patients the expression of *TMEM51, NOTCH1, EEPD1, MAFB*, and *HLA-DRB5* remained increased in monocytes of treated patients (**Figure 2B**). All these genes are remarkably specific for monocyte cluster within other PBMC clusters as well as for CD14.Mn.S100A8.9hi and CD14.Mn.MHC2hi subpopulations, but not for CD16.Mn (except HLA-DRB5) one within the whole monocyte cluster. Functional annotations confirmed the particular activation of the following pathways for NACT-treated monocytes when comparing to untreated ones: leukocyte transendothelial migration, MHC class II protein complex binding, cell adhesion molecules, TGFb signaling, cholesterol homeostasis, Wnt beta catenin signaling, and ECM binding (**Supplementary Figures S2E, F**). Interesting that *SIGLEC1* remained to be a signature gene for S100A8.9hi untreated monocytes, and *MS4A4A* – for MHC2hi untreated monocytes. It means that chemotherapy may not affect their expression. By comparing treated vs untreated cancer samples, we also found upregulation of glycolysis regulator *PFKFB3* in NACT-induced monocytes. In our recent study PFKFB3 was indicated as a prognostic biomarker for colon cancer (19). Its expression was significantly elevated in peripheral blood monocytes in colon cancer compared to rectal cancer and healthy control. PFKFB3 expression correlated to M2-polarized macrophages and indicated poor prognosis in colon cancer patients (19).

To show what affected the change of the monocyte subpopulation composition more – the tumor itself, in case of tumor-naïve samples, or the added effect of chemotherapeutic treatment we then analyzed our single-cell data comparing compositional changes of major cell types in PBMCs between cancer patients and healthy controls. The single-cell compositional data analysis (scCODA) was applied (30). In contrast to other commonly used models, scCODA proposes Bayesian approach for cell-type composition differential abundance analysis. It allows to work with low number of experimental replicates and accounts joint modeling of all measured cell-type proportions instead of individual ones. Reference cell type was set automatically to NK.CD56hi. Decreased proportions of CD14.Mn.S100A8.9hi cell subsets were observed in NACT-treated cancer patients compared to healthy controls (log2FC=0,824, FDR=0,1) (**Figure 2C**). It can be explained by their facilitated recruitment to tumor promoted by chemotherapeutic treatment (49). Despite the only slight decrease in the amount of S100A8.9hi monocytes, this fact did not diminish NACT-mediated effects.

We analyzed specific effects of NAC by comparison of NAC-treated monocytes vs. treatment-naive samples. In CD14.Mn.S100A8.9hi subset we found the upregulation of mentioned above NACT-affected genes *TMEM51, NOTCH1, EEPD1, MAFB*, and *HLA-DRB5* as well as more unique genes for this cluster *SERPINB2, SMAD6, TGFBR2, ID1, PXN, ADAM10, ADAM9, ITGAX, IL31RA, IL6R*, and *RHOU* among others. The activation of cell adhesion molecule binding, MHC class II protein complex binding, leukocyte transmigration, TGFb signaling, and other pathways was observed (**Figures 2D, E**). The expression of such genes as *HLA-DQA2, CD300LD, S100A5 LGALS1, ELOVL5, SPRED2, SLC2A3, ELOVL5, NINJ1, HMGA1*, and *E2F3* was exclusively elevated in CD14.Mn.MHC2hi monocytes of treated patients compared to untreated monocytes. Activated genes were related to the following pathways: peptide antigen assembly with MHC protein complex, leukocyte cell-cell adhesion, positive regulation of wound healing, regulation of Notch signaling pathway, regulation of cell-cell adhesion, regulation of angiogenesis, leukocyte transendothelial migration, Rap1 signaling pathway, adherens junction, and antigen processing and presentation (**Figures 2D, E**). CD16.Mn subset was less numerically significant compared to other subsets (**Figures 2D, E**).

### 3.3 Antigen presentation is a distinct feature for chemotherapy-induced monocytes

Platinum-based chemotherapy can induce immunogenic cell death (ICD) associated with tumor cell damage, leading to cell surface protein expression, cytokine secretion, or plasma membrane rupture and subsequent release of the intracellular material (50, 51). The released intracellular molecules that are damage-related molecular patterns (DAMPs) make antigen-presenting cell (APCs) including macrophages sensitive to the recognition of tumor antigens. MHC class II molecules are expressed primarily on the surface of APCs and present peptides derived from extracellular antigens (52). Monocytes/macrophages are the most abundant MHC class II positive cells in tumor microenvironment (53).

**Figure 3:**
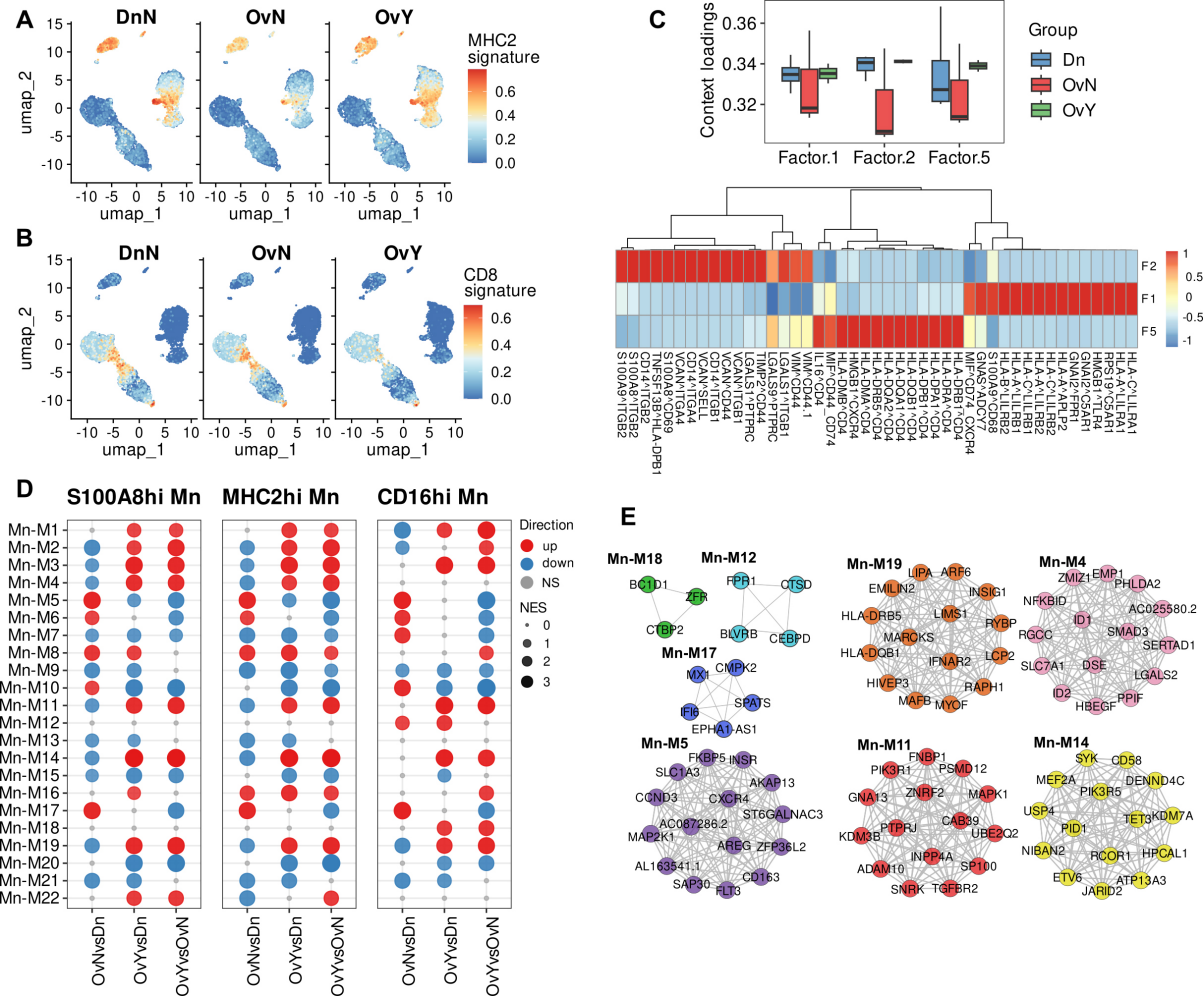
Chemotherapy induces activation of antigen presentation in peripheral blood monocytes. **(A)** NAC-related gene signature (MHC2 signature) obtained using UCell tool. **(B)** CD8-associated gene signature obtained using UCell tool **(C)** Factor loadings per each group (constructed using LIANA with Tensor-cell2cell) (Upside). Top 15 ligand-receptor pairs associated with *factor 1*, *factor 2* and *factor 5* (Underside). **(D)** Module enrichment with GSEA in individual comparison by monocyte subpopulations (CD14.Mn.S100A8.9hi; CD14.Mn.MHC2hi; CD16.Mn) (FDR<0.05). NS, not significant. OvNvsDnN – treatment-naive vs. healthy control; OvYvsDn – NAC-treated vs. healthy control; OvYvsOvN – NAC-treated vs. treatment-naive. **(E)** DEGs (FDR<0.1) reveled in distinct module-associated groups.

Surprisingly, we revealed notable and specific upregulation of genes of MHC class II protein complex, in monocytes under chemotherapy treatment, but not in untreated monocytes. These genes include *HLA-DRB5, HLA-DQA2, HLA-DQB1*, and *HLA-DQB2* (**Figure 3A**). Increased MHC class II gene expression was driven largely by classical monocytes (S100A8.9hi and MHC2hi) it was a feature of chemotherapeutic impact across all three monocyte subsets. Antigen presentation requires not only the expression of HLA genes on APCs, but also the activation of T cells (54, 55). We analyzed whether T lymphocytes which interact with APCs can be functionally affected by NACT. No remarkable changes were observed in CD4 naïve T, CD4 memory T, and Treg cells. But there were significant changes in transcriptomic profile of CD8 memory T cells. We noticed decreased expression of *IL7R, IL12RB2, IL5RA, IL21R, GZMK*, and other genes in CD8.T.EM subset (**Figure 3B**). These interleukin receptors are responsible for thymic development, T cell maturation and T-cell immune response (56–58), and their inhibition can lead to defected immune response. Downregulation of these T cell regulatory genes was observed for both NACT-treated and untreated samples, indicating that changes in CD8+ T cell transcriptome is induced by tumor-released factors and are not altered by chemotherapy (**Supplementary Figures S3A, B**).

We then directed to ligand-receptor identification which may underlie the interactions within PBMCs. To decipher cell-cell communications in PBMCs affected by NACT, we used tool integrated LIANA, a database of ligand-receptor interactions, and Tensor-cell2cell, a dimensionality reduction approach devised to uncover cell-cell communication programs across multiple samples (59). After inferring cell-cell communication with LIANA from our PBMC data, and running a Tensor Component Analysis with Tensor-cell2cell, 7 factors were obtained (**Supplementary Figure S4**), each of which represents a different cell-cell communication program. Four vectors that include the sample, ligand-receptor interaction, sender cell type, and receiver cell type were obtained for each factor (**Supplementary Figure S4**).

Applied bioinformatics tool demonstrated that *factor 5* was associated with antigen presentation via MHC class II molecules and their interactions with CD4 T cells (**Figure 3C**). We then focused on the factors where monocytes were “sender cells” or “receiver cells”. Most selectively monocytes act as the “receiver cells” within other PBMCs in *factor 1*. In-depth analysis revealed that Factor 1 is related to antigen presentation via MHC class I molecules as well as monocyte activation (**Figure 3C**). Oppositely, in *factor 2* monocytes in addition to acting as the “receiver cells”, also were the main “sender cells”. A number of cell-cell interactions, associated with adhesion molecules, were indicative of *factor 2* and likely characterized the adhesion to the vascular wall, which conforms to the DEG analysis described above. Most interesting was the fact that factor 1 and factor 2 were suppressed in untreated patients and were similar for NACT-treated samples and donors’ samples, while factor 5 related to antigen presentation via MHC class II was upregulated in NACT-induced PBMCs. This is in line with our above-mentioned results and further confirms the noticeable activation of antigen presentation via MHC class II in monocytes under chemotherapy.

### 3.4 Chemotherapy-induced antigen presentation potential in monocytes is accompanied by inhibition of interferon signaling and activation of pro-tumor monocyte polarization

Next, we analyzed gene networks provided by distinct co-expression modules built by closely correlated transcript patterns. We used hdWGCNA, a comprehensive base for analyzing co-expression networks in high-dimensional transcriptomics data (36). This bioinformatics tool allowed us to reveal 22 co-expression modules for monocytes that differed between investigated groups (**Figure 3D**). All modules contain strongly correlated hub genes. Functional annotations performed with Enrichr and names we gave to modules are indicated in **Table 1**.

**Table 1:** Module characteristics.

Module	Name	Top 5 hub-genes	Top enrichment terms
Mn-M1	Immune-regulated	*FCGR3A, CDKN1C, RHOC, HES4, SETBP1*	Neutrophile Degranulation; Complement; Fc Gamma R-Mediated Phagocytosis; Innate Immune System; Complement; B cell receptor signaling pathway; Natural killer cell mediated cytotoxicity
Mn-M2	Ribosomal protein module	*RPL26, RPS12, RPL10, RPL32, RPL28*	Cytoplasmic Translation; Peptide Chain Elongation; Formation Of A Pool Of Free 40S Subunits; GTP Hydrolysis And Joining Of 60S Ribosomal Subunit
Mn-M3	RUNX2-regulated module	*MED13L, ZEB2, KMT2C, STK10, RAPGEF1*	Transcriptional regulation by RUNX2; MET Promotes Cell Motility; Downregulation of SMAD2/3: SMAD4; Signaling By MET; Regulation Of RUNX1 Expression And Activity
Mn-M4	TGFb-related module	*RGCC, LGALS2, EMP1, ITGA5, KLF10*	TGF-beta Signaling; Positive Regulation of Extracellular Matrix Assembly; TNF-alpha Signaling via NF-kB
Mn-M5	Tumor-hormone receptors related module	*FKBP5, FMN1, PTEN, AKAP13, CD163*	PI3K-Akt signaling pathway; Hypoxia; Estrogen Response Early; Estrogen Response Late; Glycolysis; TGF-beta Signaling; Androgen Response
Mn-M6	Undefined	*GIMAP7, CD52, PCF11, PLCG2, TTC1*	No significant terms
Mn-M7	Immune defense response	*VCAN, S100A12, LYZ, DPYD, ARHGAP26*	Neutrophil Degranulation; Positive Regulation Of Response To External Stimulus; Positive Regulation Of Defense Response; Signaling By Interleukins; Hemostasis; Macrophage Activation; Complement
Mn-M8	Oxidative phosphorylation	*S100A4, TMSB10, TYROBP, SERF2, S100A6*	Oxidative phosphorylation; Respiratory Electron Transport; Proton Motive Force-Driven Mitochondrial ATP Synthesis; Citric Acid (TCA) Cycle And Respiratory Electron Transport; Cellular Respiration
Mn-M9	TNF-alpha Signaling via NF-kB	*CCDC200, FOSB, CXCL8, IL1B, ATF3*	TNF signaling pathway; NF-kappa B signaling pathway; Hypoxia; IL-17 signaling pathway; p53 Pathway; NF-kappa B signaling pathway; Toll-like receptor signaling pathway
Mn-M10	Angiogenesis-related module	*MBNL1, MEGF9, MAP3K5, VMP1, F13A1*	Complement; Negative Regulation Of Cellular Response To Vascular Endothelial Growth Factor Stimulus; Regulation Of Blood Vessel Endothelial Cell Migration; Regulation Of Platelet Activation
Mn-M11	Rho GTPases intracellular signaling	*DOCK2, PAN3, PUM2, ADAM10, CUL3*	Signaling By Rho GTPases; Ubiquitin mediated proteolysis; Class I MHC Mediated Antigen Processing And Presentation; TLR4 Cascade; Antigen Processing: Ubiquitination And Proteasome Degradation; Signaling By Receptor Tyrosine Kinases; RHO GTPase Effectors
Mn-M12	Immuno-metabolic module	*GAPDH, CAPG, CSTA, BLVRB, S100A10*	Neutrophil Degranulation; Degradation Of Cysteine And Homocysteine; Cellular Response To Chemical Stress; Sulfur metabolism; Glycolytic Process; Innate Immune System
Mn-M13	Ubiquitination and Rho GTPases	*COP1, VPS13B, USP25, ZDHHC20, PICALM*	Ubiquitin-Dependent Protein Catabolic Process; Ubiquitin mediated proteolysis; Positive Regulation of Autophagy; Signaling By Rho GTPases; RHO GTPase Effectors
Mn-M14	Immune synapsis	*JARID2, PID1, ZFAND3, RCOR1, MEF2A*	Signal Transduction; Nervous System Development; Wnt-beta Catenin Signaling; Axon Guidance; Developmental Biology; Regulation Of T Cell Activation Via T Cell Receptor Contact With Antigen Bound To MHC Molecule On Antigen Presenting Cell
Mn-M15	Endocytosis	*CELF2, MIS18BP1, SBF2, RNF144B, SCLT1*	Cytosolic Transport; Endosomal Transport; Golgi To Endosome Transport; Vesicle Fusion; Protein Ubiquitination; Endocytosis; Intracellular Protein Transport
Mn-M16	Undefined	*FTL, AIF1, TMSB4X, CST3, IFI30*	Signaling By B Cell Receptor (BCR); Mitochondrial Electron Transport, Cytochrome C To Oxygen; Beta-catenin Independent WNT Signaling; Proteasome; CLEC7A (Dectin-1) Signaling; Ubiquitin-dependent Degradation Of Cyclin D
Mn-M17	Interferon response	*MX1, IFI44L, IFI44, IFIT1, EPSTI1*	Interferon Gamma Response; Interferon Alpha Response; Defense Response To Virus; Interferon Alpha/Beta Signaling
Mn-M18	GTPase activity dependent module	*UTRN, PTPRC, FAM117B, NCOA2, ITSN2*	RAC1 GTPase Cycle; Bacterial invasion of epithelial cells; Signaling By Rho GTPases, Miro GTPases And RHOBTB3; RAC2 GTPase Cycle; RAC3 GTPase Cycle; Clathrin-mediated Endocytosis
Mn-M19	Antigen processing and presentation	*HLA-DRB1, HLA-DQA1, HLA-DQB1, HLA-DRB1, HLA-DRA*	Antigen processing and presentation; Th1 and Th2 cell differentiation; Peptide Antigen Assembly With MHC Class II Protein Complex; Cell adhesion molecules; Positive Regulation Of Leukocyte Cell-Cell Adhesion
Mn-M20	Undefined	*MARCH1, SSH2, ANKRD44, PLCL2, TNRC6B*	RAC1 GTPase Cycle; Mitotic Spindle; Class I MHC Mediated Antigen Processing And Presentation; Antigen Processing: Ubiquitination And Proteasome Degradation; Regulation Of GTPase Activity; Signaling By Rho GTPases
Mn-M21	TNF-alpha Signaling	*CD83, RASGEF1B, NR4A1, NAMPT, JUNB*	TNF-alpha Signaling via NF-kB; Apoptosis; Reactive Oxygen Species Pathway; Interferon Gamma Response; Inflammatory Response
Mn-M22	Tumor immune response	*RUNX1, TREM1, IL6R, LUCAT1, ATP2B1*	Inflammatory Response; Epithelial Mesenchymal Transition; Positive Regulation Of Collagen Metabolic Process; Hypoxia; Interleukin-4 And Interleukin-13 Signaling

We registered the most essential alterations in modules M4, M5, M7, M11, M14, M17, and M19. Among these modules, M4, M11, M14 and M19 were significantly upregulated in NACT-affected monocytes (**Figure 3D**). Module of special focus was M19 related to antigen presentation via MHC class II. *HLA-DR* and *HLA-DQ* genes formed hub gene pattern for this module (**Figure 3E**). Module 4 was characterized by multiple processes mainly related to TGFb signaling, one of the main indicator of M2 pro-tumor macrophage polarization (12). M11 was associated with Rho GTPases intracellular signaling that regulates cell adhesion and motility, as well as endocytic and exocytic vesicle trafficking (59, 60). Module 14, which we annotated as “immune synapse”, was functionally attractive. This module caught our close attention due to the significant upregulation of its core genes associated with the formation of immune synapses (61–63) in our NACT-treated samples. Less is known about the formation of such synapses between monocytes and lymphocytes, but it is critical for understanding how the presentation of tumor antigens from macrophage/monocyte to T cell can be impaired by anti-cancer treatment.

Oppositely, M17 related to interferon response was clearly suppressed in NACT-treated samples but activated in tumor untreated samples. We referred M7 to immune defense response module and it was accurately inhibited in both treated and untreated cancer samples, indicating general tumor-induced immune suppression. Different mechanisms of immune dysfunction in cancer are known (64, 65). Interesting observation was made for M5 that was assigned with processes related to the tumor-hormone receptors interactions, including estrogen and androgen responses (**Table 1**). M5 expressed differently in treated and untreated samples: increased in treatment-naïve samples and downregulated in NACT-affected ones (**Figure 3D**). Altogether these data indicated that NACT induces antigen presentation activity coming with inhibition of IFN-dependent factors, immunosuppression and increasing pro-tumor orientation.

M12 and M18 were clearly defined as CD16-spesific modules and were annotated with multiple immune regulated functions as well as with GTPase activity. According to the literature data, non-classical CD14lowCD16hi monocytes involved in the patrolling and innate local surveillance, and did not cross-present antigen to CD8+ T cells (66). Non-classical monocytes patrol the vasculature, clearing dying endothelial cells, and protecting vessel health (49, 67). Above we noticed that despite the CD16hi subset undergoing both overall tumor-dependent and NACT-specific changes, these changes are represented less extensively compared to CD14-expressing subsets (S100A8.9hi and MHC2hi).

### 3.5 Chemotherapy-activated antigen presentation potential in monocytes is not epigenetically controlled

**Figure 4:**
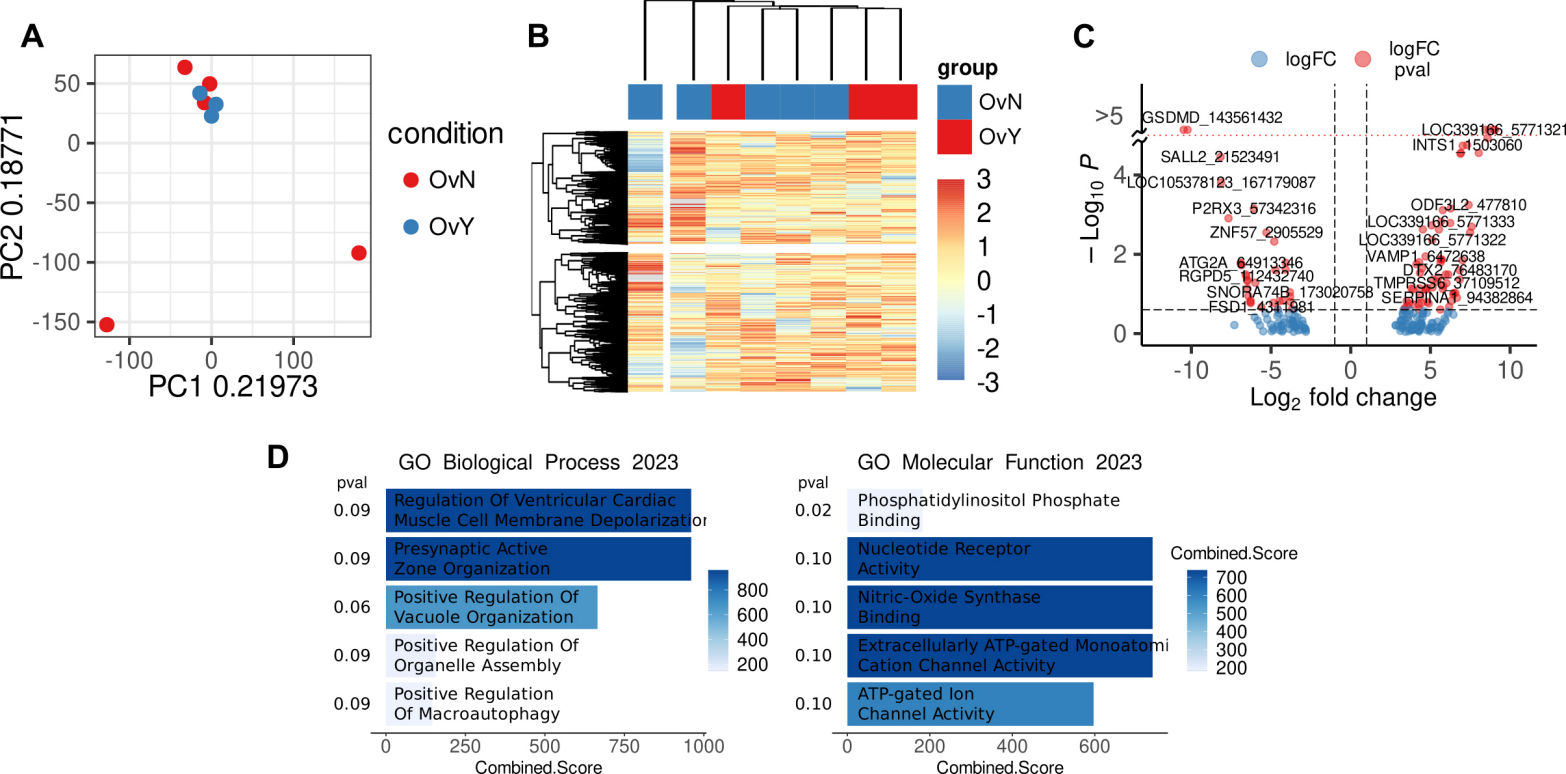
Methylation level in CpG sites in CD14+ monocytes of treated vs. untreated samples **(A)** PCA plot demonstrated analyzed samples (OvY – NAC-treated monocytes; OvN – treatment naive monocytes). **(B)** Heatmap with hierarchical clustering of OvY vs. OvN samples using top 10 000 variable CpG cites. **(C)** Volcano plot with differentially methylated CpG sites near promoter region (|L2FC|>1, FDR<0.25). **(D)** Functional annotation of genes with differentially methylated CpG sites near promoter region performed with Enrichr (FDR<0.1).

It is known that functional reprogramming of cells can be driven by synchronized regulation of gene expression, which is mediated by epigenetic modulation (68). To check whether the expression of HLA class II genes is regulated epigenetically, we performed CpG methylation analysis by reduced representation bisulfite sequencing (RRBS). RRBS was done on peripheral blood CD14+ monocytes obtained from eight HGSOC patients (n=3 for NACT-treated and n=5 for treatment-naïve) and healthy volunteers (n=10). Raw read processing and QC resulted in eight high quality samples having about 30 million raw reads each. After CpG methylation calling in Bismark there were around 385 million CpG sites detected with 40.5% mean methylation in group without NACT and 41.3% mean methylation in NACT group. Raw CpG coverage matrices from Bismark were imported in R environment and analyzed using generalized linear modeling in edgeR package. CpG sites were excluded if came from chrY and unassembled chromosomes and if had less than 8x coverage resulting in 1,303,255 CpG sites. M-values of CpG methylation was used in exploratory analysis. Top 10 000 variable CpG sites were used in principal component analysis (PCA) and hierarchal clustering (**Figures 4A, B**). Exploratory analysis did not indicate significant difference between NACT-treated and treatment-naïve samples. Differential methylation analysis revealed 67 differentially methylated CpG sites in NACT group and 33 differentially methylated CpG sites in treatment-naïve group (FDR<0.25) near promoter regions: +/- 10 kb from TSS (**Figure 4C**). Functional annotation of genes with differentially methylated CpG sites near promoter region did not indicate any biological processes observed in scRNAseq data. We also did not find methylation/demethylation of CpG sites in HLA class II or other regulators of antigen presentation or even tumor activity (**Figure 4D**). We did not find any valuable changes when we analyzed differentially methylated CpG sites in treatment-naïve vs. healthy control (**Supplementary Figures S5A–D**) and NAC-treated vs. healthy control (**Supplementary Figures S5E–G**). We concluded that chemotherapy-activated programs in monocytes were not epigenetically controlled.

## 4 Discussion

The major aim of our study was to uncover cancer specific transcriptional and epigenetic changes in peripheral blood monocytes in patients with HGSOC. Another insistent point was to unravel how chemotherapy can re-program monocytes and how it can relate to changes in other immune subpopulations in blood. Monocytes are highly plastic innate immune cells that display significant heterogeneity during homeostasis, inflammation, and tumorigenesis (69). Re-education of circulating monocytes toward T-cell stimulatory macrophages upon their extravasation in the tumor may be introduced in addition to known immunotherapeutic strategies. Identifying mechanisms capable of “re-educating” circulating monocytes will likely represent a useful strategy to prevent relapse and metastasis development after or even during anti-cancer treatment (14). However, understanding a tumor-associated monocyte profile is complicated by the fact that phenotypes of human peripheral blood monocytes display considerable heterogeneity between individuals and depending on cancer type results in uncovering subsets with differential reprogramming (14). Data on monocyte profile alterations induced by ovarian cancer-derived microenvironment are limited and require detailed analysis. The most common feature of peripheral blood monocytes of cancer patients reported in several studies is the acquisition of immunosuppressive activity and the downregulation of the MHC class II surface protein HLA-DR, a key mediator of antigen presentation (14). In present study, we performed single-cell RNA sequencing of PBMCs in patients with ovarian cancer who underwent neoadjuvant chemotherapeutic treatment and in treatment-naïve patients. Monocyte cluster was significantly affected by tumor-derived factors as well as by chemotherapeutic impact. We revealed that, in general, cancer induces the suppression of antigen presentation and the immune-inflammatory state in monocytes, but the activation of interferon-dependent pathways and pro-tumor metabolism.

Circulating monocytes are a dynamic population of cells consisting of multiple subsets that differ in phenotype, size, morphology, and transcriptional profiles (49, 69). In humans, these subsets can be distinguished by the expression of CD14 and CD16 and divided into CD14+ CD16− classical, CD14+ CD16+ intermediate and CD14-CD16+ nonclassical monocytes (49). Bioinformatical analysis allowed us to reveal three distinct monocyte subpopulations within PBMCs based on feature gene expression – CD14.Mn.S100A8.9hi, CD14.Mn.MHC2hi and CD16.Mn subsets.

Interesting observation was made in our study for CD16 cluster. Cancer-specific changes that we observed were strongly indicative for S100A8.9hi and MHC2hi subsets, but in less extend for CD16 subset. It is known that CD14+ classical monocytes represent the majority of monocytes in blood and are recruited to tissues to replenish macrophages in homeostasis and disease, whereas CD14-negative monocytes are a minority (∼7%) of human blood monocytes (70). According to existing data, non-classical CD14lowCD16hi monocytes are involved in the patrolling and innate local surveillance, they clear dying endothelial cells and protect vessel health, but do not cross-present antigen to CD8+ T cells (49, 66, 67, 70). Using tool integrated LIANA and Tensor-cell2cell we found that CD16hi monocytes have two distinct modules that are not found in CD14-expressing subsets. These modules are characterized by some immune-regulatory functions and by the regulation of cell adhesion, that corresponds to existing data.

Bioinformatical analysis helped us to reveal distinct cancer-specific features in CD14.Mn.S100A8.9hi, CD14.Mn.MHC2hi and CD16.Mn subsets. We focused on two functionally attractive for monocyte/macrophage lineage cell genes – *SIGLEC1* and *MS4A4A*. Herewith *SIGLEC1* is specifically expressed in CD14.Mn.S100A8.9hi cluster, and *MS4A4A* was significantly indicative for CD14.Mn.MHC2hi cluster within whole PBMC population. Sialic acid binding Ig like lectin 1 (Siglec1) is adhesion molecule playing role in endocytosis (71, 72). However, little is known about Siglec1 on monocytes, but minor data demonstrated that higher expression of Siglec1 on tumor-associated macrophages correlated to worse prognosis in cancer patients (17). The tetraspan surface molecule MS4A4A is specific for monocyte-macrophage lineage cells and highly expressed in TAMs. It is known that MS4A4A can promote T cell exhaustion, and is associated with poor prognosis in several cancers (73, 74). Identifying specific profile alterations in peripheral blood monocytes can serve as diagnostic, predictive, and prognostic less invasive biomarkers as well as may correlate with the efficacy of antitumor therapy (14).

The most intriguing result was NACT-induced antigen presentation by MHC class II molecules in monocytes. Increased MHC class II gene expression was a feature observed across all three monocyte subpopulations after chemotherapy. Again, despite CD16hi subset undergoing both tumor-dependent and NACT-specific changes at a direction similar to classical monocytes (S100A8.9hi and MHC2hi), these changes are represented less extensively compared to CD14-expressing subsets.

Some chemotherapeutic agents, including platinum ones, are able to drive immunogenic cell death (ICD) that is based on the release of potential immunogenic signals, known as “damage-associated molecular patterns” (DAMPs), from dying cells to induce immune responses (75–77). Presentation of tumor-associated antigens via the major histocompatibility complex (MHC) class I and II is fundamental for building a robust immune response (78). It is known that MHC class I, expressed on many cells, are generally recognized by cytotoxic CD8^+^ T cells, while MHC class II that are expressed preferentially by professional APCs, activate CD4^+^ T cells, which play critical roles in supporting CD8+ T-cell activation and generation of memory T cells (54, 78).

In tumor murine model, it was demonstrated that MHC class II^hi^ tumor-associated macrophages (TAMs) accumulated at early stages and had tumor suppressive activity, in contrast, MHC class II^low^ TAMs became more dominant in advanced tumor and supported tumor growth (79). Single-cell RNAseq data we obtained revealed that the expression of genes of MHC class II are suppressed in treatment-naïve tumor samples. But surprisingly, MHC class II expression was up-regulated after chemotherapy. Our results are in a line with the recent study where authors performed single cell RNAseq analysis using paired PBMC samples from ovarian cancer before and after NACT (80). In this study, treatment with NACT was associated with increased expression of HLA class II and antigen presentation genes on all monocyte subpopulations, although CD14++CD16– classical monocyte population was likely the major source of upregulated HLA class II within the monocyte cluster. Authors revealed increased numbers of memory T-cell receptor (TCR) clonotypes and increased central memory CD8+ and regulatory T cells after chemotherapy, however, in total NACT did not alter the composition of circulating T cells (80). Another scRNAseq analyses performed in PBMCs of one recurrent OC patient before and after NAC treatment demonstrated the tendency towards an exhaust state of CD8+ T cells under chemotherapy (81). In our study we also did not notice remarkable changes in CD4 naïve T, CD4 memory T, and Treg cells. Only in CD8 memory T cells there were decreased expression of some interleukin receptors and effector molecules that are responsible for thymic development, T cell maturation and T-cell immune response.

The success of immunotherapy, including immune checkpoint blockade (ICB) and immune cell–based immunotherapies, can depend on the effective antigen presentation to cytotoxic immune cells (54, 78). The impairments in the intercommunications between APCs and T cells can be a serious reason for the incomplete response to anti-cancer therapy (54, 55). Thus, it was shown that TAMs have the potential to phagocytose and process tumor-associated antigens, but fail to successfully support T cell activation (82). Using a fluorescent mouse model of spontaneous immunoevasive breast cancer authors identified a subset of myeloid cells that ingest tumor-derived proteins and present processed tumor antigens to reactive T cells, but do not support T cell activation or sustain cytolysis (82). Another study using lattice light sheet microscopy, demonstrated that TAMs and CD8+ T cells interact by shaping long-lasting, antigen-specific synaptic contact resulted in T cell exhaustion. Reciprocally, exhausted CD8+ T cell actively recruit monocytes to the TME and increase MHC class II expression in differentiated macrophages (62). The importance of T cell-macrophage interactions in the TME was demonstrated in one more study (83). Tumor-specific CD4 T cells instructed MHC class II-expressing monocytes to differentiate into anti-tumor macrophages (83). Furthermore, IFNγ signaling on antigen-presenting TAMs combined with cognate interaction with T cells is necessary for the most effective antitumor response (54, 84). MHC class II-expressing monocytes with high endocytic activity and IL-10 production after chemotherapy could initiate immunosuppression (85). Our data demonstrated that chemotherapy inhibited interferon-dependent signaling pathways, but activated some TGFb-related genes. According to above mentioned literature data it can be related to incomplete anti-tumor response.

All these facts demonstrate that the immune system must be equipped to detect and eliminate dying cells after chemotherapy. Ovarian cancer remains one of the few malignancies where immune checkpoint inhibitors exhibit only modest activity as monotherapy and currently has no FDA-approved indication (80, 86, 87). In recent years, ICB has been actively developing and a large number of clinical trials are underway (86–89). However, ICB often does not show a lasting positive response and, thus, has not entered routine use in clinical practice (90). It is hypothesized that OC may be somewhat resistant to ICB due to a low intrinsic tumor immunogenicity and high mutational burden, as well as excessive immunosuppressive mechanisms in the tumor microenvironment (91). Some clinical studies indicated that NACT may be a promising platform for building combinatorial immunotherapy strategies in ovarian cancer (48, 80). Moreover, search for NACT-mediated mechanisms can be crucial for choosing the ideal timing (“window” period) providing the best opportunity for immunotherapy combined with chemotherapy (92). We believe that our study can help unveil how we can re-educate monocytes toward T-cell-stimulatory macrophages upon extravasation in the tumor and how we can educate the tumor microenvironment in ovarian cancer to respond to mono- or combination chemo-/immunotherapy.

## Data Availability

The datasets generated during the current study are available as records in the NCBI GEO repository under the following accession numbers: GSE264489 (single-cell RNAseq) and GSE264488 (RRBS).
