# Supplementary material for: Platinum-based chemotherapy promotes antigen presenting potential in monocytes of patients with high-grade serous ovarian carcinoma
Source: Front Immunol. 2024 Sep 9;15:1414716. doi: 10.3389/fimmu.2024.1414716 (PMC11417001; doi:10.3389/fimmu.2024.1414716)
Supplement: Supplementary file 1 [file DataSheet1.docx]

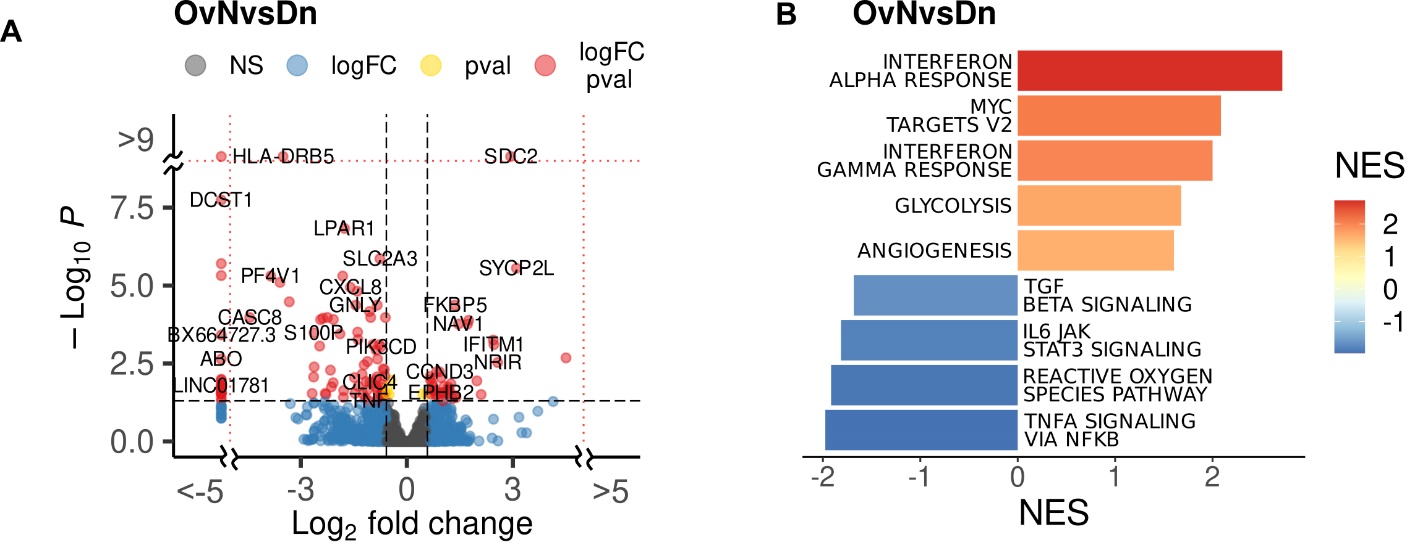


**Supplemental Figure S1.** Results of DEG analysis for combined monocyte cluster from treatment-naive (OvN) and healthy control (Dn) groups. **(A)** Volcano plot demonstrates p-value and log2fold-change value for DEGs in combined monocyte cluster versus healthy control (|L2FC|>0.58, FDR<0.05). **(B)** Bar plot with GSEA results indicates top deregulated pathways in combined monocyte cluster versus healthy control (FDR<0.1). OvNvsDnN – treatment-naive vs. healthy control.


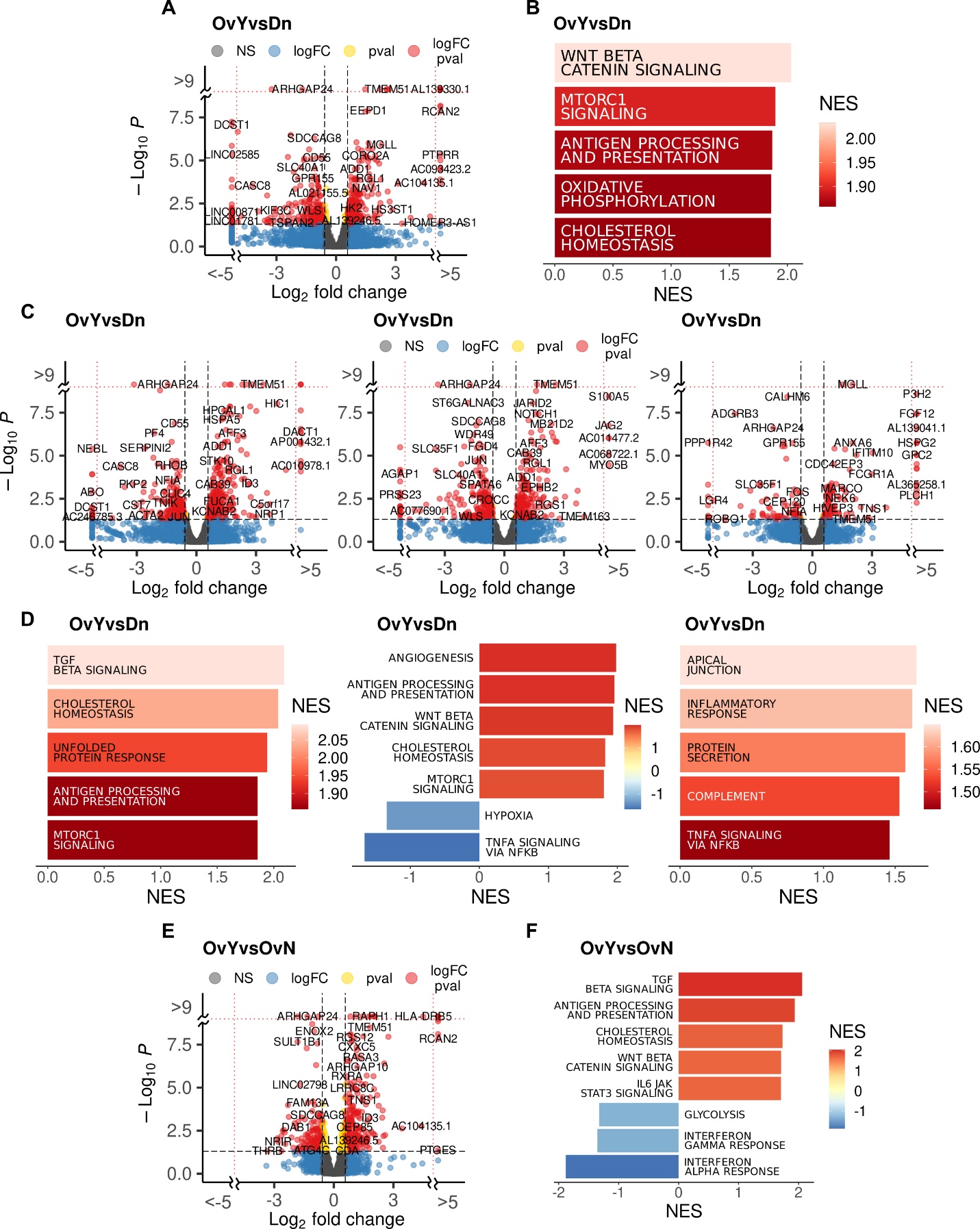


**Supplemental Figure S2.** Chemotherapy-induced transcriptional programming in whole monocyte cluster and monocyte subpopulations. **(A)** Volcano plot demonstrates p-value and log2fold-change value for DEGs in whole NAC-treated monocyte cluster versus healthy control (|L2FC|>0.58, FDR<0.05). **(B)** Bar plot with GSEA results indicates top deregulated pathways in whole NAC-treated monocyte cluster versus healthy control (FDR<0.1). **(C)** Volcano plots demonstrate p-value and log2fold-change value for DEGs in each NAC-treated monocyte subpopulation (CD14.Mn.S100A8.9hi; CD14.Mn.MHC2hi; CD16.Mn) versus healthy control (|L2FC|>0.58, FDR<0.05). **(D)** Bar plots with GSEA results indicate top deregulated pathways in each NAC-treated monocyte subpopulation (CD14.Mn.S100A8.9hi; CD14.Mn.MHC2hi; CD16.Mn) versus healthy control (FDR<0.1). **(E)** Volcano plot demonstrates p-value and log2fold-change value for DEGs in whole NAC-treated monocyte cluster versus treatment-naive (|L2FC|>0.58, FDR<0.05). **(F)** Bar plot with GSEA results indicates top deregulated pathways in whole NAC-treated monocyte cluster versus treatment-naive (FDR<0.1). OvYvsDn – NAC-treated vs. healthy control; OvYvsOvN – NAC-treated vs. treatment-naive.


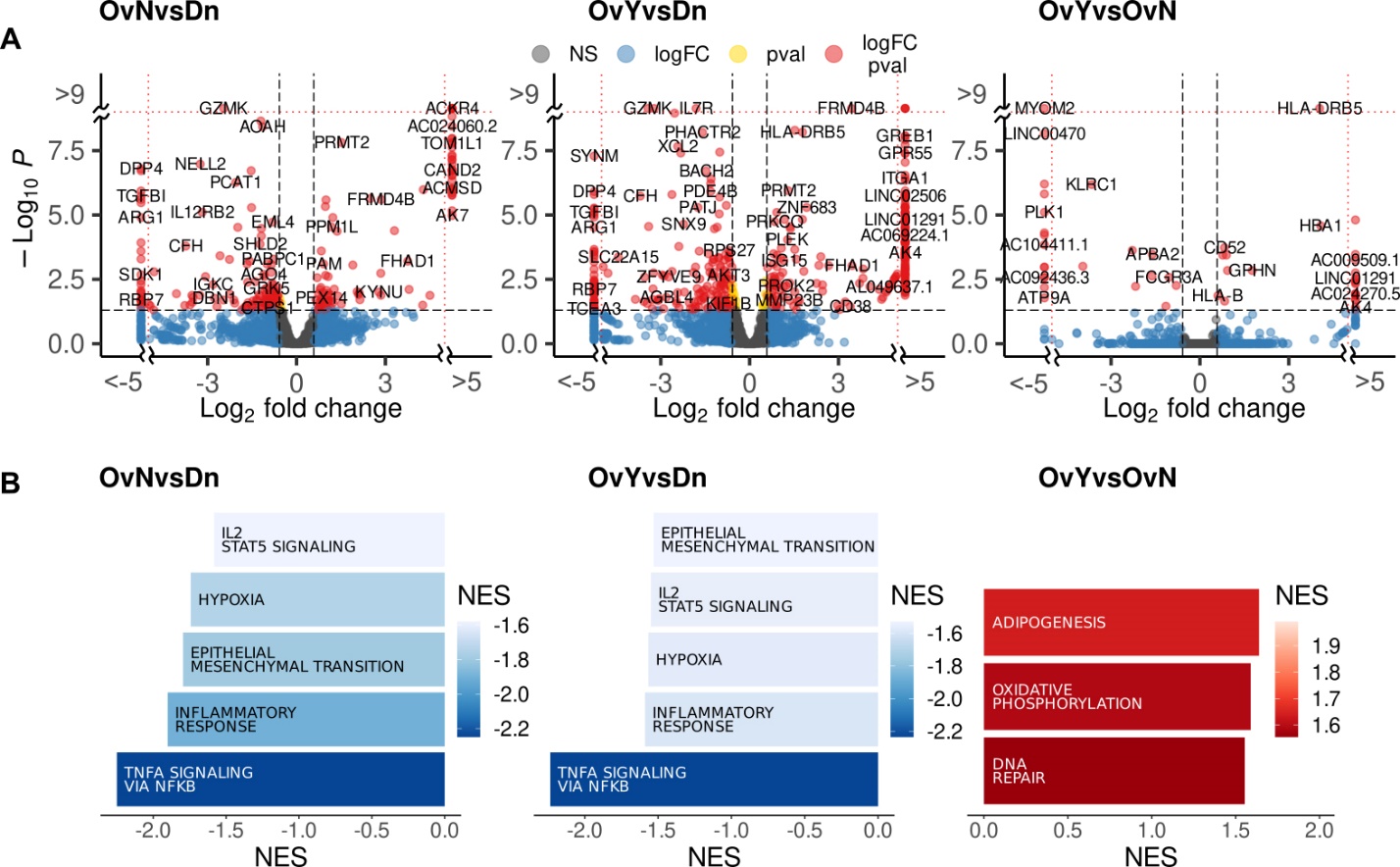


**Supplemental Figure S3.** Chemotherapy-induced transcriptional changes in CD8 T effector/memory cells. **(A)** Volcano plots demonstrate p-value and log2fold-change value for DEGs in CD8 T effector/memory cells in each comparison groups (|L2FC|>0.58, FDR<0.05). **(B)** Bar plots with GSEA results indicate top deregulated pathways in CD8 T effector/memory cells in each comparison groups (FDR<0.1). OvNvsDnN – treatment-naive vs. healthy control; OvYvsDn – NAC-treated vs. healthy control; OvYvsOvN – NAC-treated vs. treatment-naive.


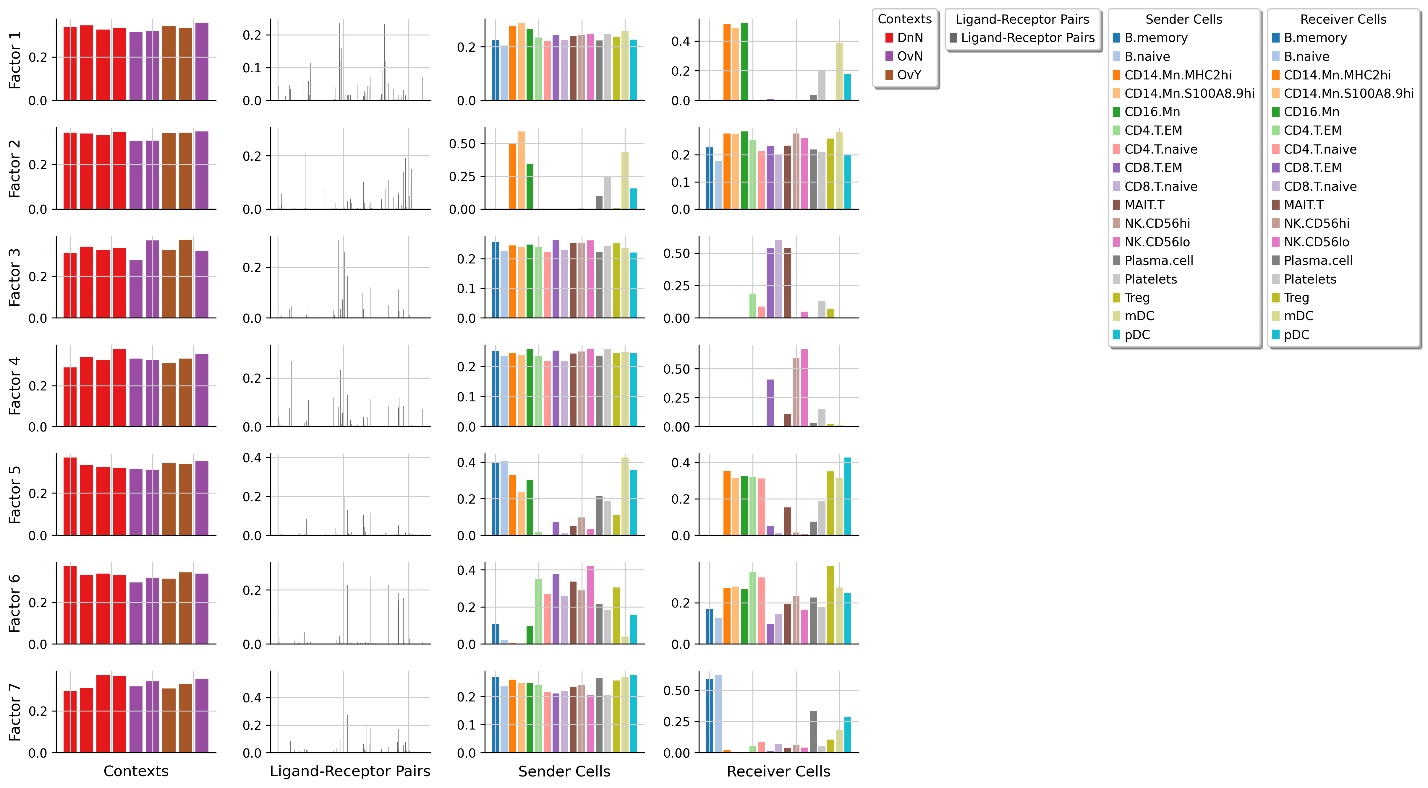


**Supplemental Figure S4.** LIANA and Tensor-cell2cell results depicting factor loadings for each sample, ligand-receptor interaction, sender cell type, and receiver cell type.


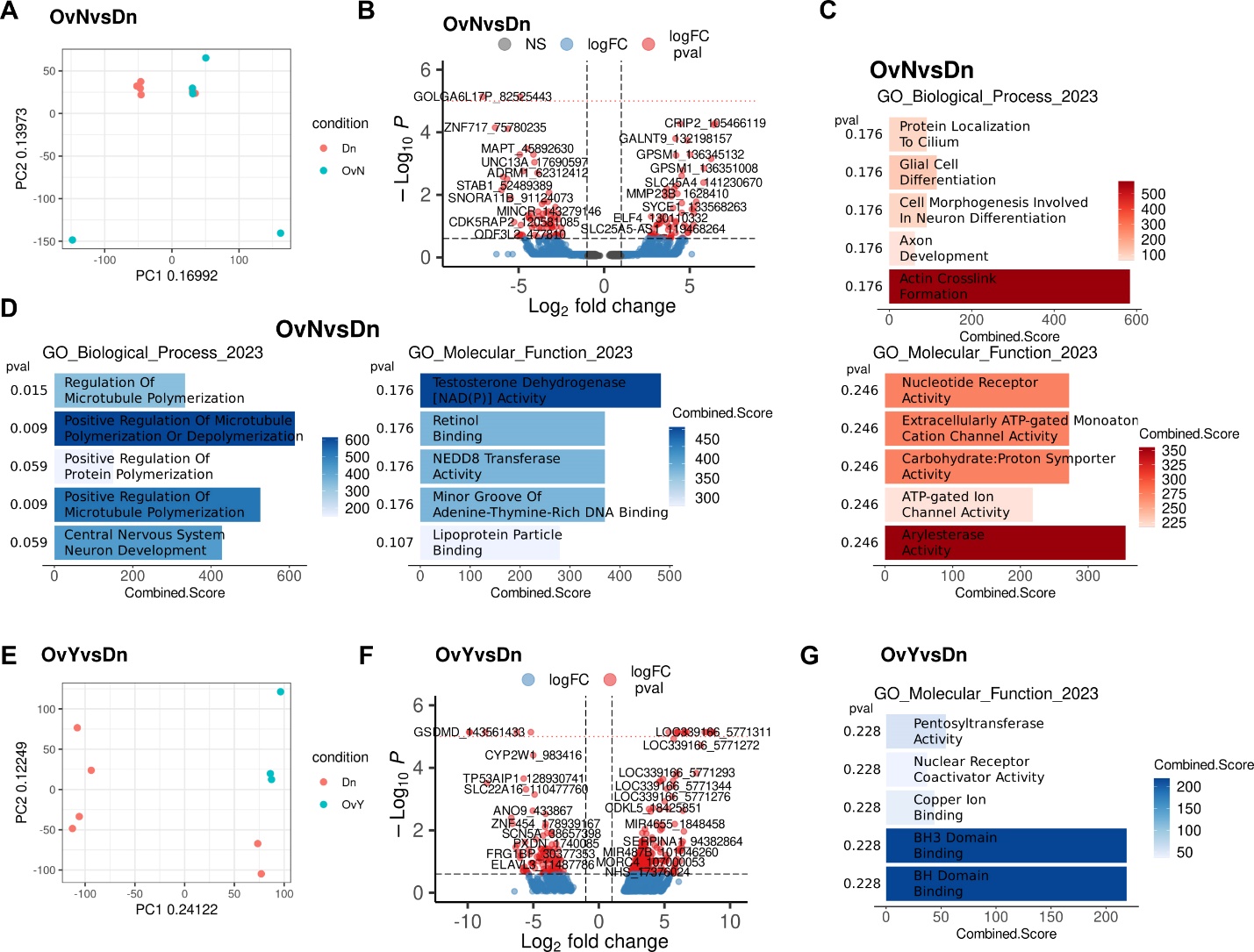


**Supplemental Figure S5. Methylation level in CpG sites in CD14+ monocytes of cancer patients versus healthy donors. (A)** PCA plot demonstrates analyzed samples (OvN – treatment naive monocytes, Dn – healthy control). **(B)** Volcano plot with differentially methylated CpG sites near promoter region (|L2FC|>1, FDR<0.25) in treatment-naïve versus healthy donors. **(C)** Functional annotation of genes with differentially hypermethylated CpG sites near promoter region performed with Enrichr (FDR<0.25) in treatment-naïve versus healthy donors. **(D)** Functional annotation of genes with differentially hypomethylated CpG sites near promoter region performed with Enrichr (FDR<0.25) in treatment-naïve versus healthy donors. **(E)** PCA plot demonstrated analyzed samples (OvY – NAC-treated monocytes, Dn – healthy control). **(F)** Volcano plot with differentially methylated CpG sites near promoter region (|L2FC|>1, FDR<0.25) in NAC-treated monocytes versus healthy donors. **(G)** Functional annotation of genes with differentially hypomethylated CpG sites near promoter region performed with Enrichr (FDR<0.25) in NAC-treated monocytes versus healthy donors. OvNvsDnN – treatment-naive vs. healthy control; OvYvsDn – NAC-treated vs. healthy control.
